# Author Correction: TRIM28-mediated nucleocapsid protein SUMOylation enhances SARS-CoV-2 virulence

**DOI:** 10.1038/s41467-026-68634-7

**Published:** 2026-02-05

**Authors:** Jiang Ren, Shuai Wang, Zhi Zong, Ting Pan, Sijia Liu, Wei Mao, Huizhe Huang, Xiaohua Yan, Bing Yang, Xin He, Fangfang Zhou, Long Zhang

**Affiliations:** 1 The Eighth Affiliated Hospital, Sun Yat-sen University, Shenzhen, 518033 China; 2 Institutes of Biology and Medical Sciences, Soochow University, Suzhou, 215123 China; 3 MOE Key Laboratory of Biosystems Homeostasis & Protection and Innovation Center for Cell Signaling Network, Life Sciences Institute, Zhejiang University, Hangzhou, 310058 China; 4 Shenzhen Key Laboratory of Systems Medicine for Inflammatory Diseases, School of Medicine, Shenzhen Campus of Sun Yat-sen University, Shenzhen, 518107 China; 5 International Biomed-X Research Center, Second Affiliated Hospital of Zhejiang University, Zhejiang University School of Medicine, Hangzhou, 310058 China; 6 Zhejiang Hospital, Zhejiang University School of Medicine, Hangzhou, 310058 China; 7 Faculty of Basic Medical Sciences, Chongqing Medical University, Chongqing, 400016 China; 8 Department of Biochemistry and Molecular Biology, School of Basic Medical Sciences, Nanchang University, Nanchang, 330031 China; 9 Department of Pharmaceutical Chemistry and the Cardiovascular Research Institute, University of California, San Francisco, CA 94158 USA; 10 Institute of Human Virology, Zhongshan School of Medicine, Sun Yat-sen University, Guangzhou, 510080 China

Correction to: *Nature Communications* 10.1038/s41467-023-44502-6, published online 04 January 2024

The original version of this Article contained an error in Figure 4h due to a copy-paste error. The correct version of Figure 4h is (correct images labelled in blue):
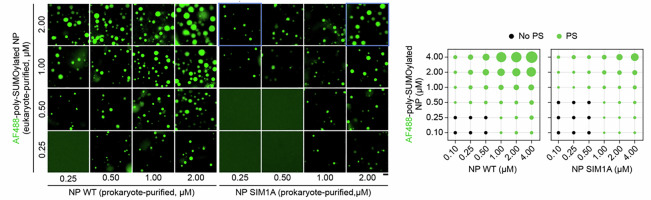


which replaces the previous incorrect version (incorrect images labelled in red):
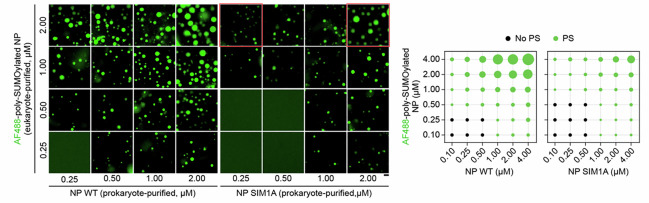


This has now been corrected in both the PDF and HTML versions of the Article.

