# Supplementary material for: TRIM28-mediated nucleocapsid protein SUMOylation enhances SARS-CoV-2 virulence
Source: Nat Commun. 2024 Jan 4;15:244. doi: 10.1038/s41467-023-44502-6 (PMC10764958; doi:10.1038/s41467-023-44502-6)
Supplement: Supplementary file 1 — Supplementary Information [file 41467_2023_44502_MOESM1_ESM.pdf]

Supplemental information

TRIM28-mediated nucleocapsid protein SUMOylation enhances SARS-CoV-2 virulence

This file includes:

Supplementary Fig. 1 to 10 and figure legends

Supplementary Table 1: The human or mouse primer sequences for qPCR

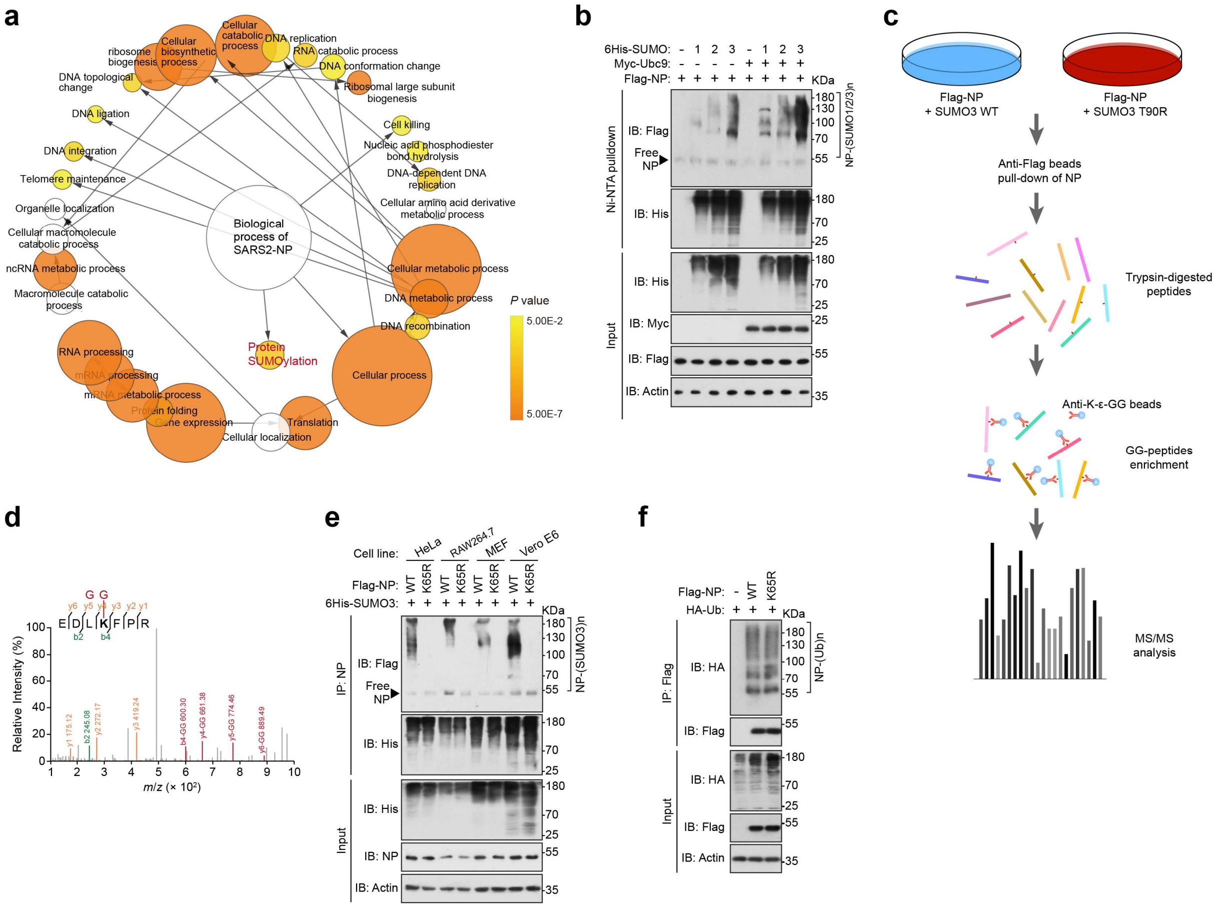

Supplementary Fig. 1: Related to Fig. 1; SARS2-NP can be modified by poly-SUMO.

a Interactive graph of the enriched GO terms under the 'biological process' category, which is derived from SARS2-NP interactome after pull down of NP in the SARS-CoV-2-infected A549 cells stably expressing hACE2 (A549-hACE2). The arrows represent the orientation relation between class and subclass of biological process. **b** SUMOylation assay. Immunoblot (IB) of total lysates (input) and Ni-NTA pulldown of cell lysates from HEK293T cells transfected with indicated expression plasmids. **c** Schematic representation of the method used for mapping of NP SUMOylation site. HEK293T cells were co-transfected with SUMO3 WT/T90K and Flag-NP. SARS2-NP were then enriched by anti-Flag beads pull-down and subsequently digested by trypsin, which generates a mixture of modified and nonmodified peptides. Under these conditions, identification of modified peptides is challenging, because they represent a very small fraction of the total amount of peptides. Peptides containing GG-K were enriched by anti-K-ε-GG beads and analyzed by tandem mass spectrometry (MS/MS). **d** Mass spectrum of a SARS2-NP peptide modified at K65 by SUMO3 T90R. **e** SUMOylation assay. IB of total lysates and Ni-NTA pulldown of cell lysates from HeLa, or Raw264.7, or MEF, or Vero E6 cells transfected with indicated expression plasmids. **f** Ubiquitination assay of SARS2-NP. IB

of total lysates and anti-Flag IP from HEK293T cells transfected with indicated expression plasmids. Data are representative of at least two (**d**) or three (**b**, **e**, **f**) independent experiments with similar results.

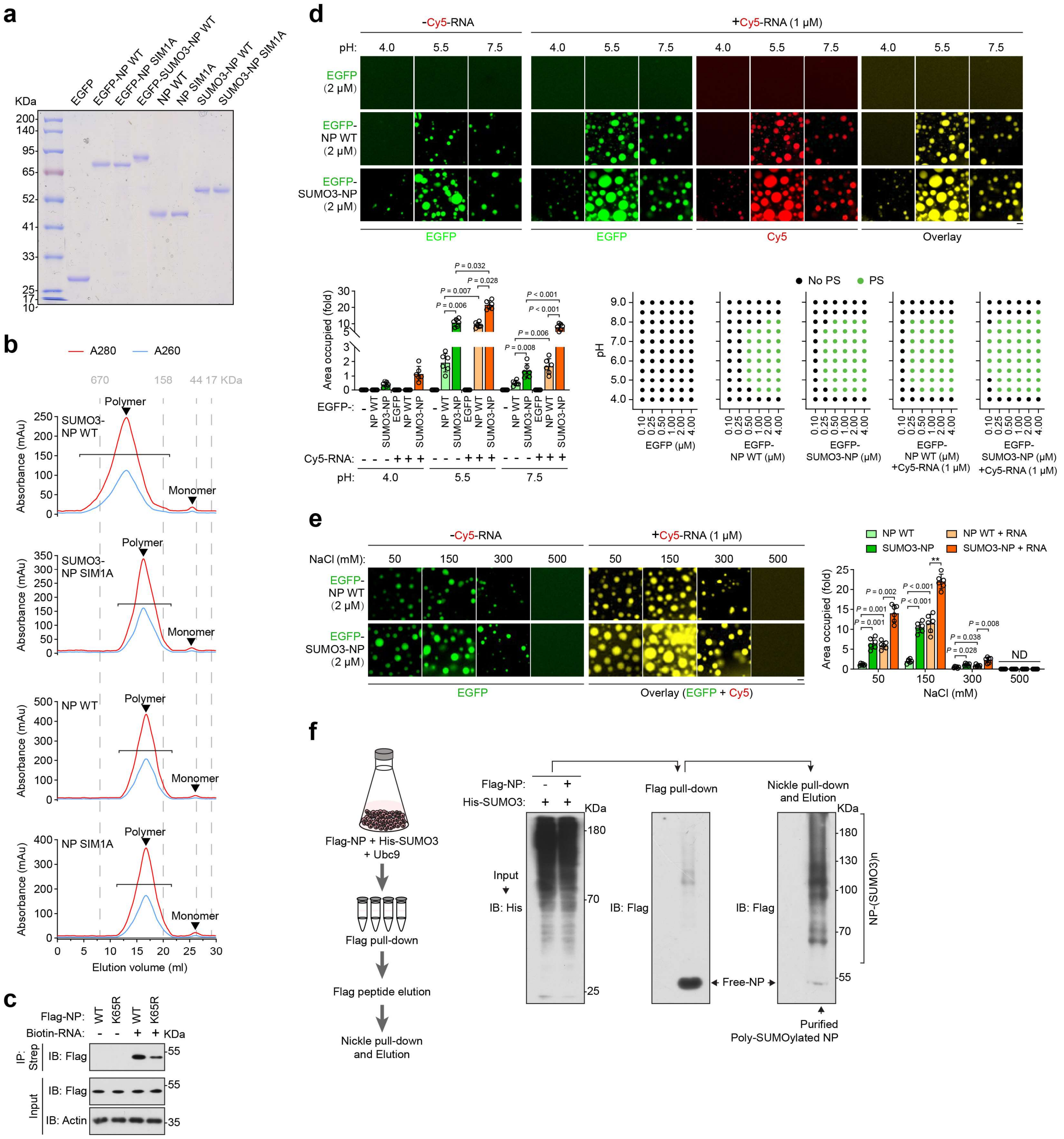

Supplementary Fig. 2: Related to Fig. 2; Enhanced LLPS ability of SARS2-NP is mediated by poly-SUMOylation of SARS2-NP.

a EGFP, EGFP-SARS2-NP WT/SIM1A, EGFP-SUMO3-SARS2-NP WT, SARS2-NP WT/SIM1A, and SUMO3-SARS2-NP WT/SIM1A proteins purified from bacteria were analyzed by Coomassie blue stain of SDS-PAGE. **b** Size-exclusion chromatography elution profiles of prokaryote-purified SUMO3-SARS2-NP WT/SIM1A or SARS2-NP WT/SIM1A using AKTA system. Brackets represent range of multimers. Vertical dash lines represent peak elution volumes of gel filtration protein calibration standards. **c** Immunoblot of total lysates (input) and streptavidin RNA pull-down of Flag-SARS2-NP WT/K65R (IP) from HEK293T

cells transfected with indicated expression plasmids. **d** Droplet formation of prokaryote-purified EGFP, or EGFP-SARS2-NP WT, or EGFP-SUMO3-SARS2-NP WT without or with Cy5-RNA, at indicated pH. Top, representative images. Bottom-left, fold change in droplet formation; Data points indicate the relative area occupied by droplets. Bottom-right, phase separation (PS) diagram of proteins at indicated concentrations and pH values. The green dots indicate PS. And the black dots indicate no PS. **e** Droplet formation of prokaryote-purified EGFP-SARS2-NP WT or EGFP-SUMO3-SARS2-NP WT without or with Cy5-RNA, at indicated NaCl. Left, representative images. Right, fold change in droplet formation; Data points indicate the relative area occupied by droplets. **f** Left, schematic showing the steps involved in the purification of poly-SUMOylated SARS2-NP from HEK293F cells. Right, immunoblots showing purified poly-SUMOylated SARS2-NP. Data are representative of at least three independent experiments with similar results (**b**–**e**). Data are presented as Mean ± SD; n = 6 independent samples (**d** bottom-left, D right); statistical analyses were performed using one-way ANOVA. Scale bar, 10 μm(**d**, **e**).

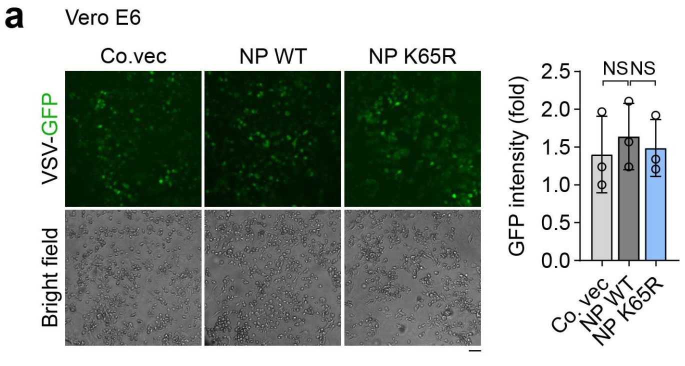

Supplementary Fig. 3: Related to Fig. 3; Loss of SUMOylation largely impairs SARS2-NP's promotion of viral replication.

a Fluorescence microscopy and bright-field of VSV-GFP in Vero E6 cells transfected with Co. vec, or SARS2-NP WT/K65R plasmids, followed by infection with VSV-GFP (m.o.i. of 0.1) for 12 h (left). Scale bar, 100 μm. The fold change in VSV-GFP intensity was quantified (right). Data are representative of at least three independent experiments with similar results. Data are presented as Mean ± SD. n = 3 independent images. Statistical analyses were performed using one-way ANOVA.

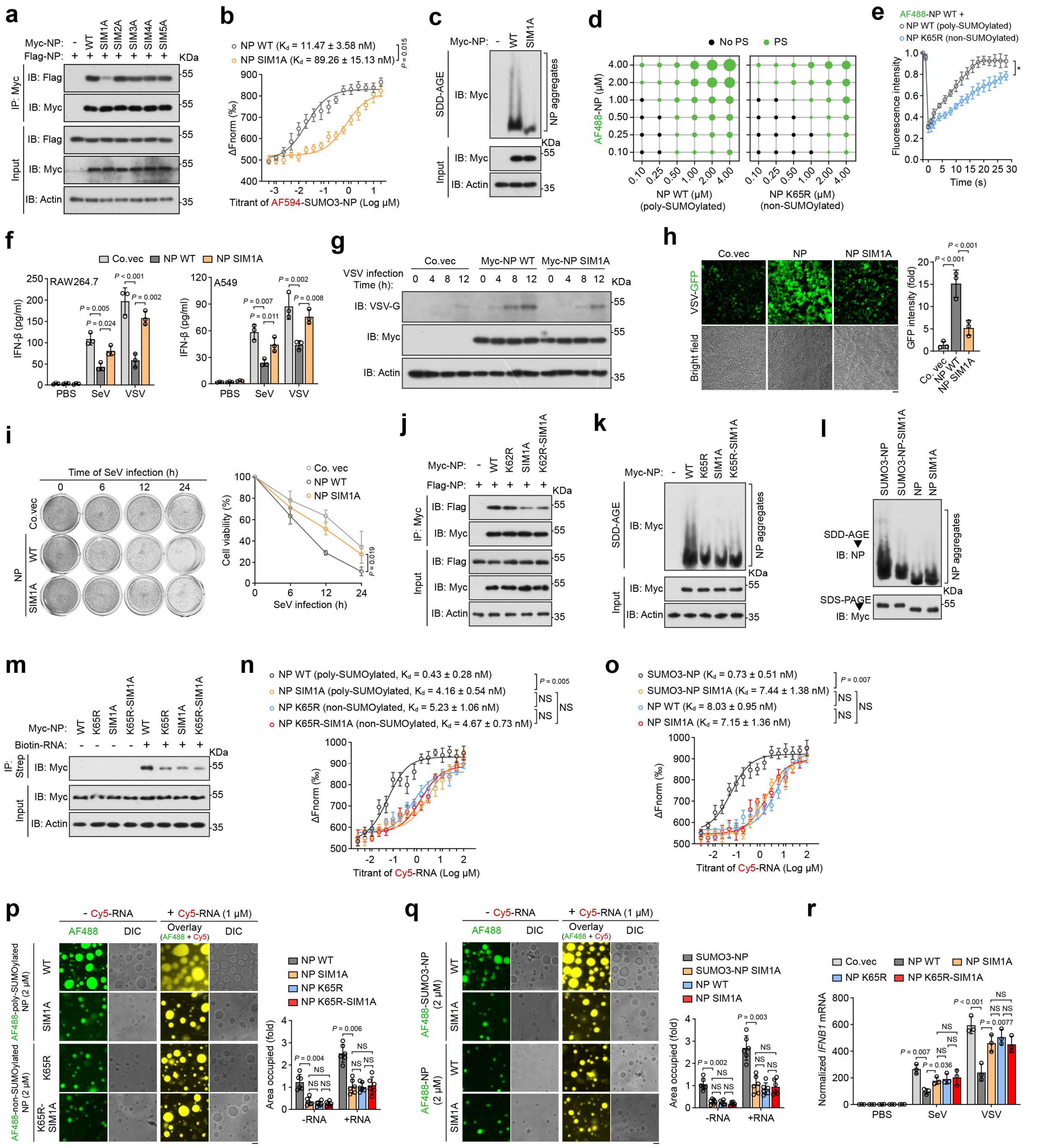

Supplementary Fig. 4: Related to Fig. 4; SUMOylation and SUMO-interacting motif (SIM) of SARS2-NP mediates self-interaction and dampens IFN-β signaling.

a Immunoblot (IB) of total lysates (input) and anti-Myc immunoprecipitates (IP) from HEK293T cells transfected with indicated expression plasmids. **b** MST assay between AF594-labeld SUMO3-SARS2-NP and SARS2-NP WT/SIM1A. All are purified from bacteria. Data points indicate the difference in normalized fluorescence (Fnorm, ‰). Curves indicate the calculated fits. **c** Aggregation assay. SDD-AGE (top) and SDS-PAGE (bottom) of lysates from HEK293T cells transfected with indicated expression plasmids. **d** Phase separation (PS)

diagram of different concentrations of AF488-labeled SARS2-NP (prokaryote-purified) with SARS2-NP WT/K65R purified from HEK293F cells. The green dots and dot sizes indicate PS, and the black dots indicate no phase separation. **e** FRAP assay of mixture of AF594-labeled SARS2-NP (prokaryote-purified) and SARS2-NP WT/K65R purified from HEK293F cells. Quantification of FRAP before and after (over a 30 s time course) photobleaching is shown. **f** Determination of the IFN-β levels in RAW264.7 (left) and A549 (right) cells transfected with the indicated plasmids and infected with SeV/VSV for 12 h. **g** Immunoblot (IB) of VSV glycoprotein (VSV-G) in MEF cells transfected with indicated expression plasmids and infected with VSV at m.o.i. of 0.1 for the indicated time periods. **h** Fluorescence microscopy and bright-field of VSV-GFP in HEK293T cells transfected with indicated expression plasmids, followed by infection with VSV-GFP at m.o.i. of 0.1 for 12 h (left). Right, the fold change in VSV-GFP intensity. **i** SeV-induced cytopathic effect (CPE) in A549 cells after being transfected with indicated expression plasmids, followed by infection with SeV for the indicated time periods (left). Right, the rates of CPE were quantified. **j** IB of total lysates and anti-Myc IP from HEK293T cells transfected with indicated expression plasmids. **k**, **l** Aggregation assay. SDD-AGE (top) and SDS-PAGE (bottom) of lysates from HEK293T cells transfected with indicated expression plasmids (**k**), or of prokaryote-purified SUMO3-SARS2-NP WT/SIM1A, or SARS2-NP WT/SIM1A (**l**). **m** IB of total lysates and streptavidin RNA pull-down of Myc-SARS2-NP WT/K65R/SIM1A/K65R-SIM1A (IP) derived from HEK293T cells transfected with the indicated plasmids. **n**, **o** MST assay between ligand Cy5-RNA and SARS2-NP WT (poly-SUMOylated)/SIM1A (poly-SUMOylated, but loss of SIM site)/K65R (non-SUMOylated)/K65R-SIM1A double mutant (non-SUMOylated, and loss of SIM site) purified from HEK293F cells (**n**), or SUMO3-SARS2-NP WT/SIM1A, or SARS2-NP WT/SIM1A purified from bacteria (**o**). **p**, **q** Droplet formation of AF488-labeled SARS2-NP WT/SIM1A/K65R/K65R-SIM1A purified from HEK293F cells (**p**), or SUMO3-SARS2-NP WT/SIM1A, or SARS2-NP WT/SIM1A purified from bacteria (**q**), without or with Cy5-RNA. Left, representative images. Right, fold change in droplet formation. **r** Normalized *IFNB1* mRNA in HEK293T cells transfected with indicated expression plasmids, followed by SeV/VSV infection for 12 h. All data are representative of at least three independent experiments with similar results. Data are presented as Mean ± SD, n = 3 (**b**, **e**, **f**, **i**, **n**, **o**, **r**, **h**) or 6 (**p**, **q**) independent samples. Statistical analyses were performed using One-way ANOVA (**f**, **h**, **p**–**r**) or Two-way ANOVA (**b**, **e**, **i**, **n**, **o**). Scale bar, 10 μm.

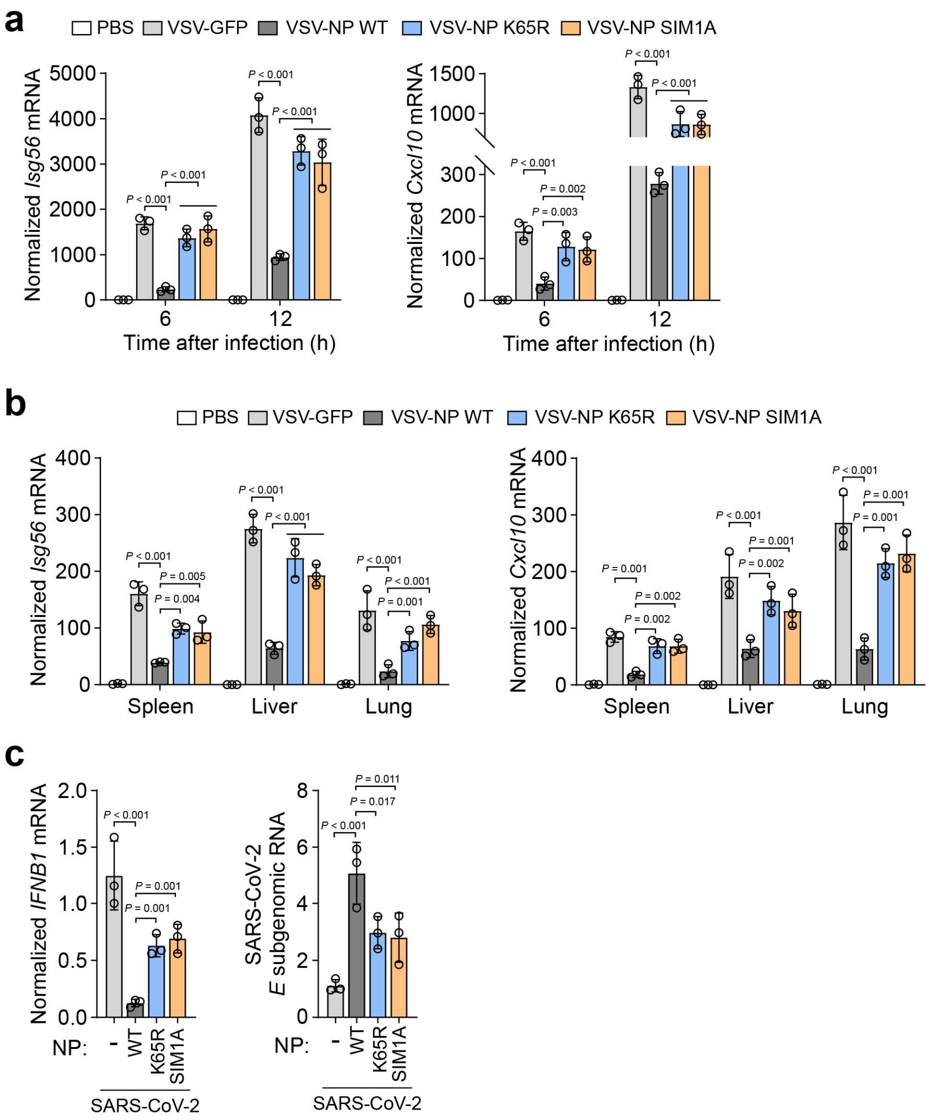

Supplementary Fig. 5: Related to Fig. 5; SUMOylation and SIM site of SARS2-NP are required for SARS2-NP-mediated robust antiviral immunosuppression.

a Normalized *Isg56* (left) and *Cxcl10* (right) mRNA in mice peritoneal macrophages infected with indicated recombinant VSV at m.o.i. of 0.1, or not. **b** Normalized *Isg56* (left) and *Cxcl10* (right) mRNA in the spleen, liver, and lungs of mice from Fig. 5d. **c** Normalized *IFNB1* mRNA (left) and SARS-CoV-2 *E* genomic RNA (right) levels in Caco-2 cells transfected with the indicated plasmids and then infected with SARS-CoV-2 for 12 h. All data are representative of at least two (**b**) or three (**a**, **c**) independent experiments with similar results. Data are presented as Mean ± SD; n = 3 independent mice (**a**, **b**) or samples (**c**). Statistical analyses were performed using a One-way ANOVA.

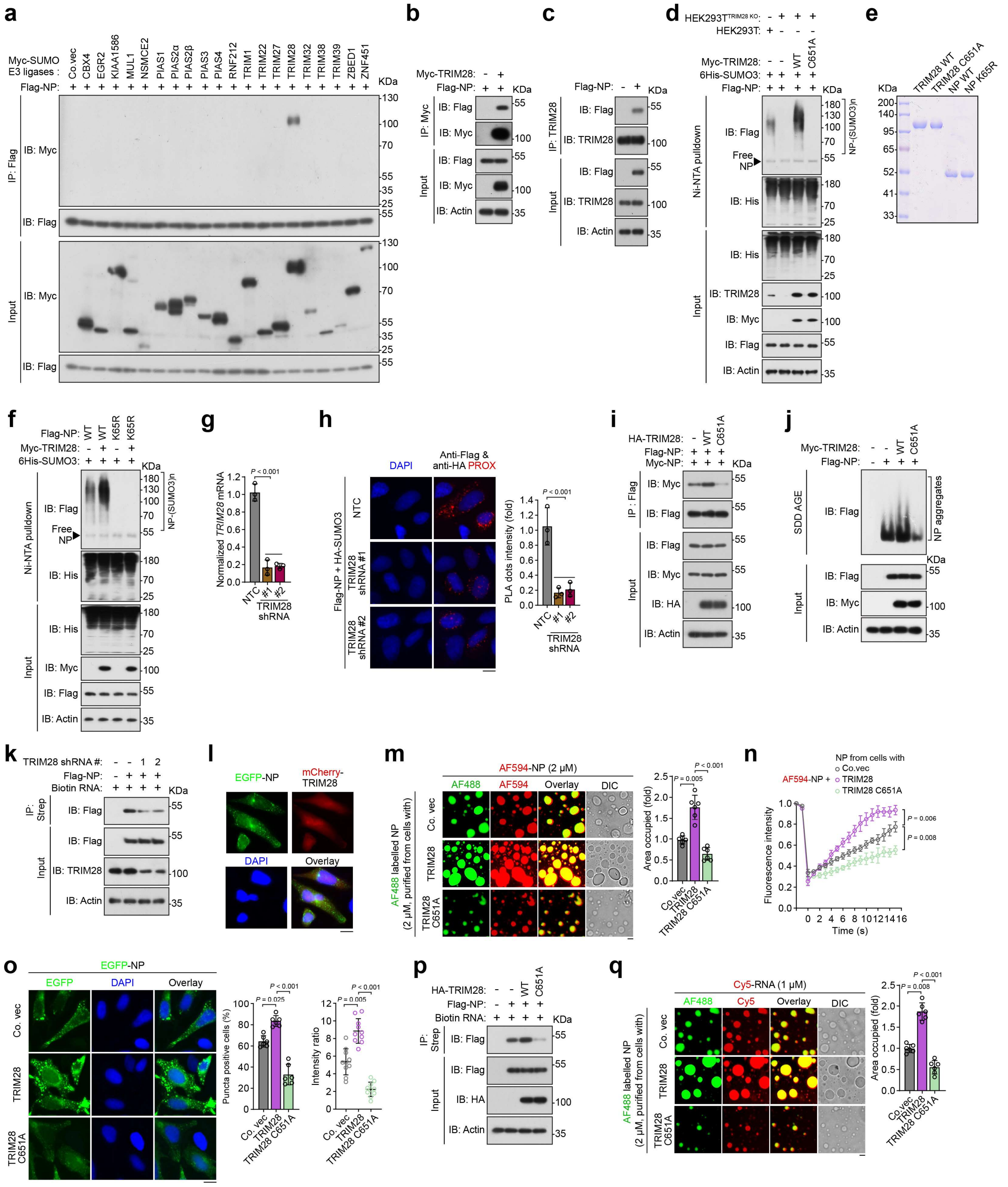

Supplementary Fig. 6: Related to Fig. 6; TRIM28 overexpression promotes SARS2-NP SUMOylation and LLPS.

a, **b**, **c** Immunoblot (IB) of total lysates (input) and anti-Flag (**a**), or anti-Myc (**b**), or anti-TRIM28 (**c**) immunoprecipitates (IP) from HEK293T cells transfected with indicated expression plasmids. **d** SUMOylation assay. IB of total lysates and Ni-NTA pulldown of cell lysates from HEK293T or TRIM28 knockout (KO)-HEK293T cells transfected with indicated

expression plasmids. **e** HA-TRIM28 WT/C651A, or SARS2-NP WT/K65R proteins purified from bacteria was analyzed by Coomassie blue stain of SDS-PAGE. **f** SUMOylation assay. IB of total lysates and Ni-NTA pulldown of cell lysates from HEK293T cells transfected with indicated expression plasmids. **g** Normalized *TRIM28* mRNA in HEK293T cells transfected with non-target control (NTC) or TRIM28 shRNA. **h** *In situ* PLA for HA-SUMO3 and Flag-SARS2-NP in HeLa cells with/without TRIM28 knockdown as indicated. Left, the PROX complexes are represented by the red fluorescent dots. Right, changes in PROX dots intensity. **i** IB of total lysates and anti-Flag IP from HEK293T cells transfected with indicated expression plasmids. **j** Aggregation assay. SDD-AGE (top) and SDS-PAGE (bottom) of lysates from HEK293T cells transfected with indicated expression plasmids. **k** IB of total lysates and streptavidin RNA pull-down of Flag-SARS2-NP derived from HEK293T cells with/without TRIM28 knockdown. **l** Co-expression of EGFP-SARS2-NP and mCherry-TRIM28 in HeLa cells. Nuclei were visualized by DAPI staining. **m** Droplet formation of a mixture of AF594-labeled SARS2-NP purified from bacteria and AF488-labeled SARS2-NP purified from HEK293F cells. Right, fold change in droplet formation. **n** FRAP assay of mixture of AF594-labeled SARS2-NP purified from bacteria and SARS2-NP purified from HEK293F cells with Co. vec or TRIM28 WT/C651A. **o** Puncta formation of EGFP-SARS2-NP in HeLa cells with Co. vec or TRIM28 WT/C651A. Left, representative fluorescence images. Middle, the percentages of cells harboring puncta. Right, the ratio of puncta-like fluorescence intensity. **p** IB and streptavidin RNA pull-down of Flag-SARS2-NP derived from HEK293T cells with Co. vec or HA-TRIM28 WT/C651A. **q** Droplet formation of a mixture of AF488-labeled SARS2-NP purified from HEK293F cells with Co. vec or TRIM28 WT/C651A and Cy5-RNA. Right, fold change in droplet formation. All data are representative of at least three independent experiments with similar results. Data are presented as Mean ± SD (**g**, **h**, **m**–**o**, **q**). n = 3 (**g**, **n**, **h**), or 6 (**m**, **o** middle, **q**), or 10 (**o** right) independent samples. Statistical analyses were performed using a One-way ANOVA (**g**, **h**, **m**, **o**, **q**) or Two-way ANOVA (**n**). Scale bar, 10 μm (**h**, **l**, **m**, **o**, **q**). DIC, differential interference contrast microscopy (**m**, **o**).

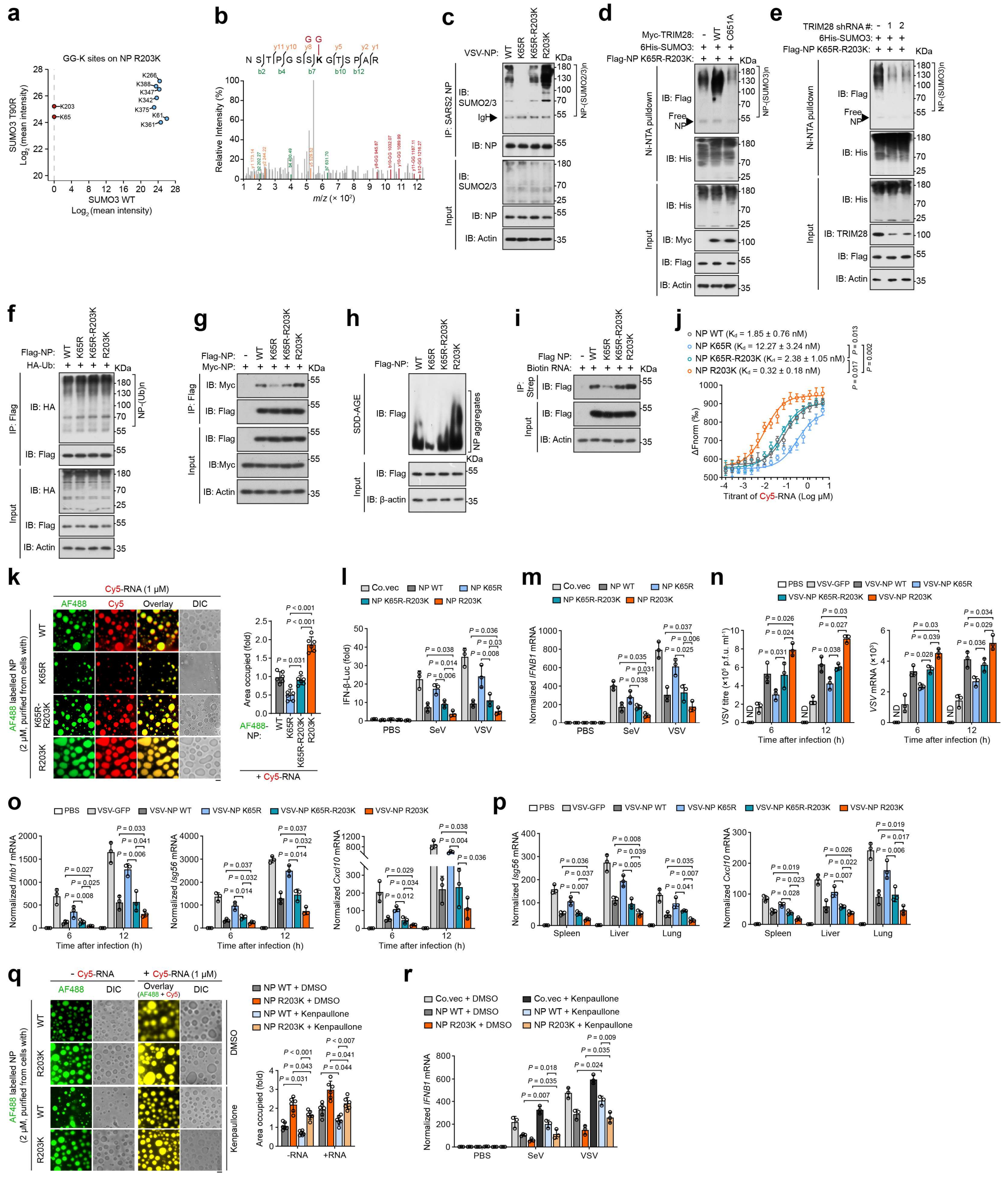

Supplementary Fig. 7: Related to Fig. 7; Natural R203K mutant of SARS2-NP further inhibits innate antiviral immunity due to gain of an extra SUMOylation.

a The mean intensity (label-free quantification) of GG-K sites of SARS2-NP R203K identified by MS/MS in SUMO3 WT/T90K-expressing HEK293T cells, as illustrated in Fig. 1c and supplementary Fig. 1c. **b** Mass spectrum of a SARS2-NP R203K peptide modified at K203 by SUMO3 T90R. **c** SUMOylation assay. Immunoblot (IB) of total lysates and anti-SARS2-NP immunoprecipitates (IP) of cell lysates from VSV-NP WT/K65R/K65R-R203K/R203K-

infected HEK293T cells. **d**–**e** SUMOylation assay. IB of total lysates (input) and Ni-NTA pulldown of cell lysates from HEK293T cells transfected with indicated expression plasmids. **f** Ubiquitination assay. IB of total lysates and anti-Flag IP from HEK293T cells transfected with indicated expression plasmids. **g** IB of total lysates and anti-Flag IP from HEK293T cells transfected with indicated expression plasmids. **h** Aggregation assay. SDD-AGE (top) and SDS-PAGE (bottom) of lysates of HEK293T cells transfected with indicated expression plasmids. **i** IB of total lysates and streptavidin RNA pull-down (IP) of Flag-SARS2-NP WT/K65R/K65R-R203K/R203K derived from HEK293T cells transfected with the indicated expression plasmids. **j** MST assay between ligand Cy5-RNA and SARS2-NP WT/K65R/K65R-R203K/R203K purified from HEK293F cells. **k** Droplet formation of a mixture of AF488-labeled SARS2-NP WT/K65R/K65R-R203K/R203K purified from HEK293F cells and Cy5-RNA. Right, fold change in droplet formation. DIC, differential interference contrast microscopy. **l** Fold change in IFN-β-luciferase (Luc) activity in HEK293T cells transfected with indicated expression plasmids, followed by SeV/VSV infection for 12 h. **m** Normalized *IFNB1* mRNA in HEK293T cells transfected with indicated expression plasmids, followed by SeV/VSV infection for 12 h. **n** VSV titres (left) and copy number (right) in mice peritoneal macrophages infected with indicated recombinant VSV at m.o.i. of 0.1, or not. ND, not determined. **o** Normalized *Ifnb1* (left), *Isg56* (middle) and *Cxcl10* (right) mRNA in mice peritoneal macrophages infected with indicated recombinant VSV at m.o.i. of 0.1, or not. **p** Normalized *Isg56* (left) and *Cxcl10* (right) mRNA in the spleen, liver, and lungs of mice form Fig. 7f. **q** Droplet formation of AF488-labeled SARS2-NP WT/R203K purified from HEK293F cells (treated without or with 0.5 μM of Kenpaullone), without or with Cy5-RNA. Left, representative images. Right, fold change in droplet formation. **r** Normalized *IFNB1* mRNA expression in HEK293T cells transfected with indicated expression plasmids and treated without or with 0.5 μM of Kenpaullone, followed by SeV/VSV infection for 12 h. Data are representative of at least two (**a**, **b**, **o**, **p**) or three (**c**–**n**, **q**, **r**) independent experiments with similar results. Data are presented as Mean ± SD (**j**–**r**). n = 3 (**j**, **l**–**n**, **r**) or 6 (**k**, **q**) independent samples, or 3 independent mice (**o**, **p**). Statistical analyses were performed using a One-way ANOVA (**k**–**q**) or Two-way ANOVA (**j**). Scale bar, 10 μm (**k**, **q**).

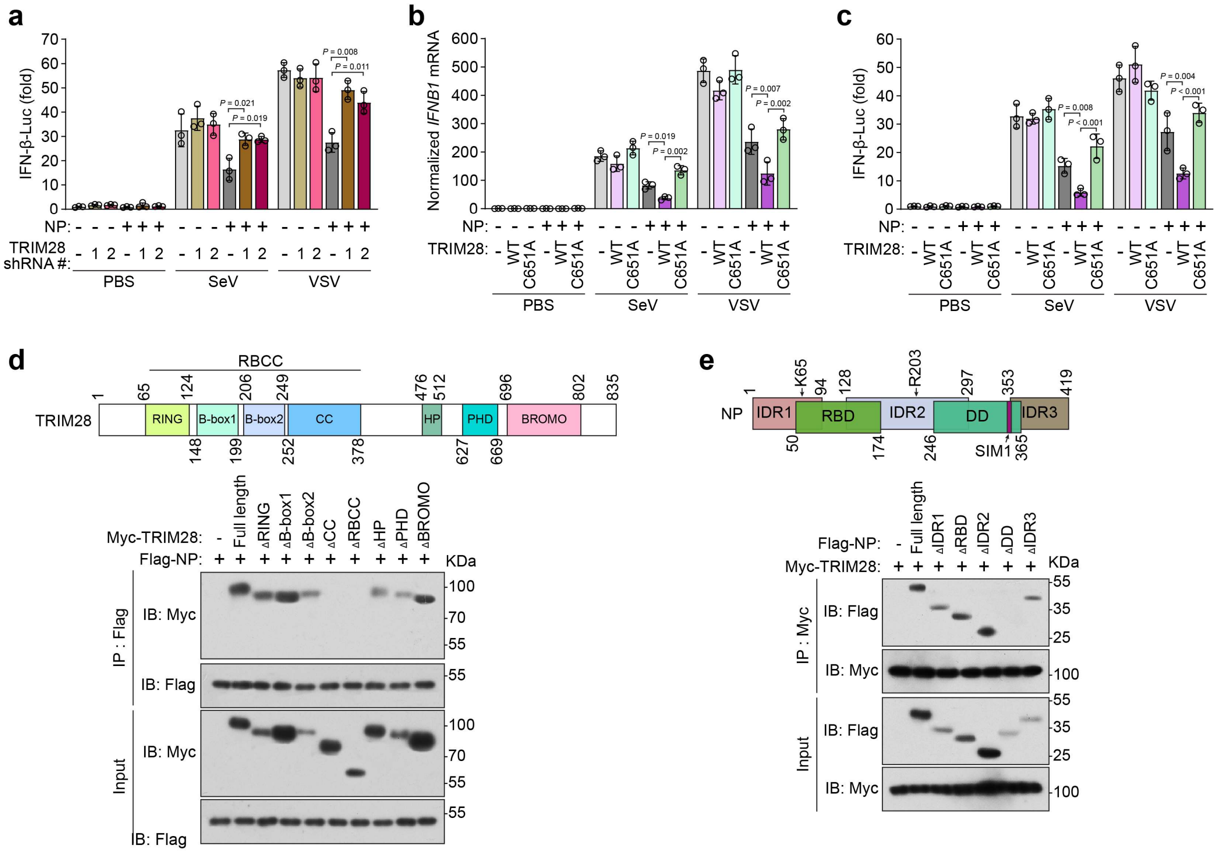

Supplementary Fig. 8: Related to Fig. 8; Manipulating TRIM28 expression affects SARS2-NP-mediated innate immune suppression.

a, **c** Fold change in IFN-β-luciferase (Luc) activity in HEK293T cells transfected with indicated expression plasmids, followed by SeV/VSV infection for 12 h. **b** Normalized *IFNB1* mRNA in HEK293T cells transfected with indicated expression plasmids, followed by SeV/VSV infection for 12 h. **d** Top, domain structure of TRIM28. RING, really interesting new gene; CC, coiled coil; RBCC, RING domain followed by B-boxes and CC domain; HP, heterochromatin protein; PHD, plant homeodomain; BROMO, bromodomain. Bottom, immunoblot (IB) of total lysates (input) and anti-Flag immunoprecipitates (IP) from HEK293T cells transfected with indicated expression plasmids. **e** Top, domain structure of SARS2-NP. IDR, intrinsically disordered region; RBD, RNA-binding domain; DD, dimerization domain. Bottom, IB of total lysates and anti-Myc IP from HEK293T cells transfected with indicated expression plasmids. All data are representative of at least three independent experiments with similar results. Data are presented as Mean ± SD; n = 3 independent samples. Statistical analyses were performed using a One-way ANOVA. Δ, deletion mutants.

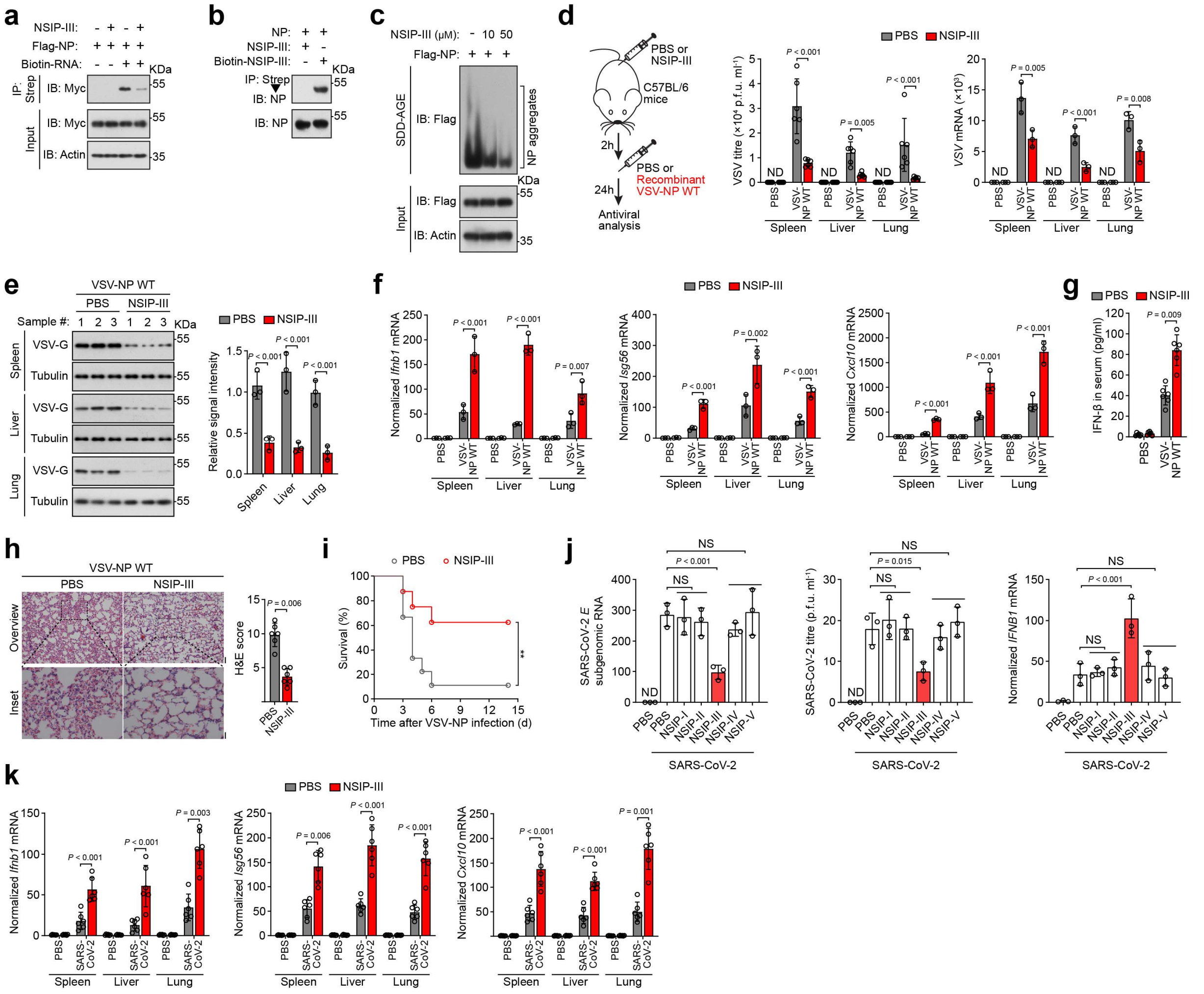

Supplementary Fig. 9: Related to Fig. 8; SARS2-NP-mediated inhibition of innate antiviral immunity is alleviated significantly by a peptide, NSIP-III.

a Immunoblot (IB) of total lysates and streptavidin RNA pull-down (IP) from HEK293T cells transfected with the plasmids expressing Flag-SARS2-NP, followed by 10 μM of NSIP-III treatment for 12 h. **b** Binding assay of NSIP-III and NP. Prokaryote-purified SARS2-NP was incubated with NSIP-III labelled without or with biotin. Streptavidin pull-down was then performed. **c** Aggregation assay. SDD-AGE (top) and SDS-PAGE (bottom) of lysates of HEK293T cells transfected with plasmids expressing Flag-SARS2-NP, followed by 10 or 50 μM of NSIP-III treatment for 12 h, or not as indicated. **d** C57BL/6 mice were pretreated with PBS or NSIP-III (25 mg/kg, i.p., 6 mice each group) for 2 h and challenged with VSV-NP WT (5×10^{8} p.f.u. per mouse, i.p) for another 24 h (left). Then, VSV titres (middle) and copy number (right) in the spleen, liver, lungs of mice were measured. **e** IB of VSV-G in the spleen, liver and lungs of mice from **d**. Right, the relative band intensity. **f** Normalized *Ifnb1* (left), *Isg56* (middle) and *Cxcl10* (right) mRNA in the spleen, liver and lungs of mice from **d**. **g** IFN-β concentration

in the serum of mice from **d**. **h** H&E staining of lung sections of mice from **d** (left). Right, the cumulative H&E score for quantification of lung lesions. Scale bars, 500 μm (left-top) and 100 μm (left-bottom). **i** Kaplan–Meier survival analysis of mice (n= 6 mice each group) pretreated with PBS or NSIP-III (25 mg/kg, i.p.) for 2 h and then challenged with VSV-NP WT (1×10^9 p.f.u. per mouse, i.p.). **j** CaCo-2 cells were pretreated PBS or NSIP-I–V (50 μM) for 2 h and infected with SARS-CoV-2 for 12 h. Left, the subgenomic *E* gene. Middle, the SARS-CoV-2 titres. Right, normalized *IFNB1* mRNA. **k** Normalized *Ifnb1* (left), *Isg56* (middle) and *Cxcl10* (right) mRNA in the spleen, liver and lungs of mice from Fig. 8h. Data are representative of at least two (**d**–**j**) or three (**a**, **b, c, k**) independent experiments with similar results. Data are presented as Mean ± SD (**d**–**h**, **j, k**). n = 3 (**d**–**f**) or 6 independent mice (**g**–**i, k**), or 3 independent samples (**j**). Statistical analyses were performed using a two-tailed Student's *t*-test (**d**–**h**, **j, k**), or log-rank test (**i**). Scale bar, 500 μm (**h** left-top), or 100 μm (**h** left-bottom). ND, not determined.

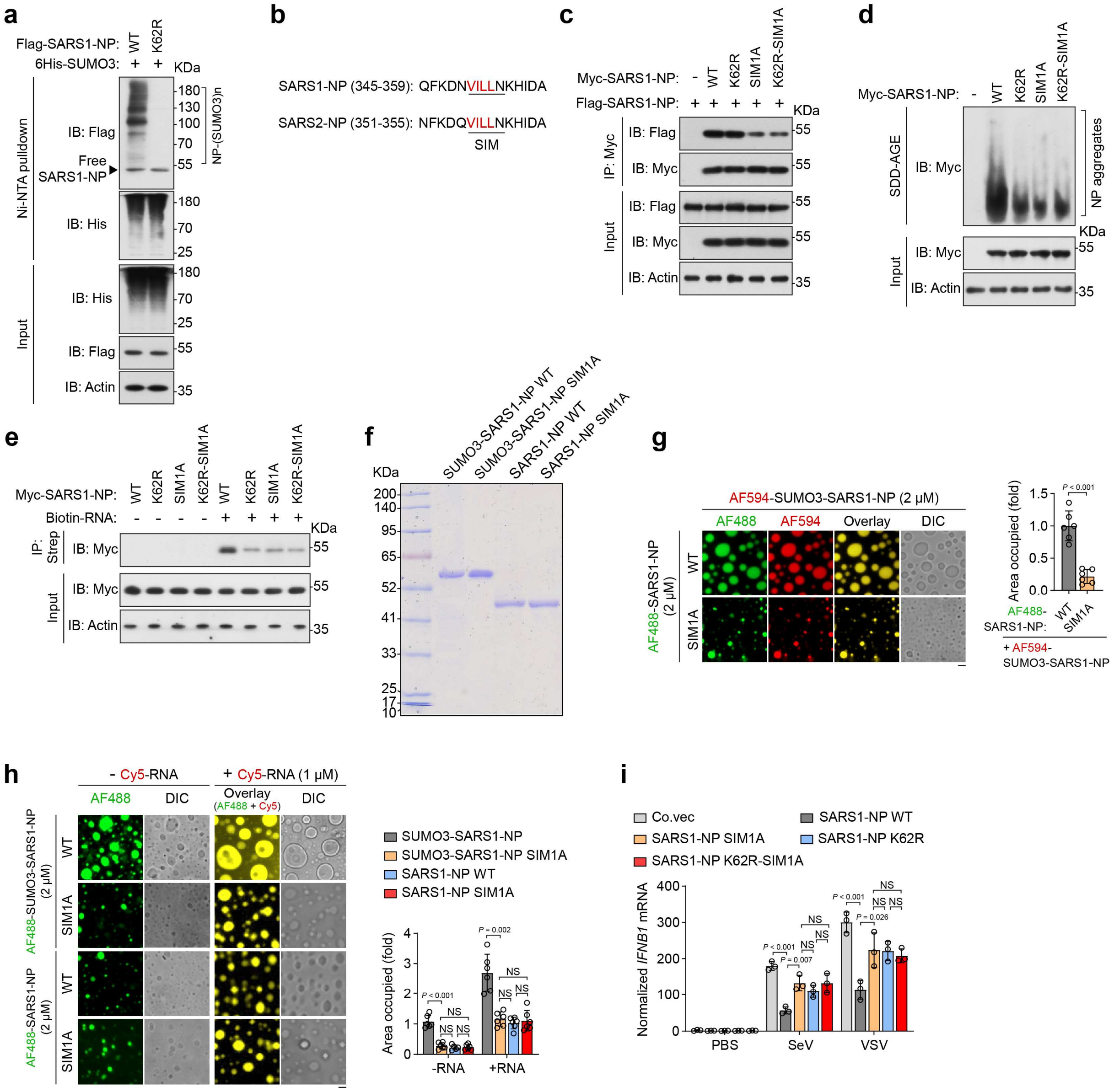

Supplementary Fig. 10: SUMO:SIM-mediated multivalent self-interaction of SARS1-NP is also implicated in SARS1-NP.

a Immunoblot (IB) of total lysates and Ni-NTA pulldown of cell lysates from HEK293T cells transfected with plasmids expressing 6His-SUMO3, plus Flag-SARS1-NP WT/K62R as indicated. **b** Sequence alignment of a conserved SUMO-interacting motif (SIM) in SARS1-NP and SARS2-NP. The indicated amino acids in red (V/I/L) within the SIM were mutated to alanine (A) in the mutants SARS1-NP SIM1A. **c** Immunoblot (IB) of total lysates (input) and anti-Myc immunoprecipitates (IP) from HEK293T cells transfected with plasmids expressing Flag-SARS1-NP plus Myc-SARS1-NP WT/K62R/SIM1A/K62R-SIM1A, or not as indicated. **d** Aggregation assay. SDD-AGE (top) and SDS-PAGE (bottom) of lysates from HEK293T cells transfected with plasmids expressing Myc-SARS1-NP WT/K62R/SIM1A/K62R-SIM1A, or

not as indicated. **e** IB of total lysates and streptavidin RNA pull-down of Myc-SARS1-NP WT//K62R/SIM1A/K62R-SIM1A (IP) derived from HEK293T cells transfected with the indicated plasmids. **f** SUMO3-SARS1-NP WT/SIM1A, SARS1-NP WT/SIM1A proteins purified from bacteria were analyzed by Coomassie blue stain of SDS-PAGE. **g** Droplet formation of AF488-labeled SARS1-NP WT/SIM1A with AF594-labeled SUMO3-SARS1-NP. All are prokaryote-purified, 2 μM each (left). Right, fold change in droplet formation; Data points indicate the relative area occupied by droplets per image field (40 ×). **h** Droplet formation of 2 μM of AF488-labeled SUMO3-SARS1-NP WT/SIM1A, or SARS1-NP WT/SIM1A purified from bacteria, without or with 1 μM of Cy5-RNA, at pH 5.5, 150 mM NaCl. Left, representative images. Right, fold change in droplet formation; Data points indicate the relative area occupied by droplets per image field (40 ×). **i** Normalized *IFNB1* mRNA expression (determined by qPCR) in HEK293T cells transfected with Co. vec, or SARS1-NP WT/SIM1A/K65R/K65R-SIM1A expression plasmids, followed by SeV/VSV infection for 12 h. All data are representative of at least three independent experiments with similar results (**a**, **c**–**e**, **g**–**i**). Data are presented as Mean ± SD (**g-i**), n = 3 independent samples (**i**), or 6 independent images (**g**, **h**). Statistical analyses were performed using Student's *t*-test (**g**), or One-way ANOVA (**h**, **i**). Scale bar, 10 μm.

Supplementary Table 1: The human or mouse primer sequences for qPCR

Gene	Sequence (5′ >>> 3′)	
Murine *Ifnb1*	Forward	TCCTGCTGTGCTTCTCCACCACA
	Reverse	AAGTCCGCCCTGTAGGTGAGGTT
Murine *Cxcl10*	Forward	ATCATCCCTGCGAGCCTATCCT
	Reverse	GACCTTTTTTGGCTAAACGCTTTC
Murine *Isg56*	Forward	AAGACAAGGCAATCACCCTCTACT
	Reverse	GTCTTTCAGCCACTTTCTCCAAA
Murine *Gapdh*	Forward	GGCCTTCCGTGTTCCTACC
	Reverse	AGCCCAAGATGCCCTTCAGT
Human *IFNB1*	Forward	CCAACAAGTGTCTCCTCCAAAT
	Reverse	AATCTCCTCAGGGATGTCAAAGT
Human *CXCL10*	Forward	TTTGCTGCCTTATCTTTCTGACT
	Reverse	ATTGTAGCAATGATCTCAACACG
Human *ISG56*	Forward	GCTTTCAAATCCCTTCCGCTAT
	Reverse	CTTGGCCCGTTCATAATTTTTC
Human *GAPDH*	Forward	AGGGCTGCTTTTAACTCTGGT
	Reverse	CCCCACTTGATTTTGGAGGGA
VSV	Forward	ACGGCGTACTTCCAGATGG
	Reverse	CTCGGTTCAAGATCCAGGT
SARS-CoV-2 *E* subgenomic RNA	Forward	CTTTCGTGGTATTCTTGCTAGTT
	Reverse	CACGTTAACAATATTGCAGCA
