# Supplementary material for: TRIM28-mediated nucleocapsid protein SUMOylation enhances SARS-CoV-2 virulence
Source: Nat Commun. 2024 Jan 4;15:244. doi: 10.1038/s41467-023-44502-6 (PMC10764958; doi:10.1038/s41467-023-44502-6)
Supplement: Supplementary file 3 — Description of Additional Supplementary Files [file 41467_2023_44502_MOESM3_ESM.docx]

**Description of Additional Supplementary Files**

**Supplementary Data 1:** SARS-CoV-2 nucleocapsid protein-interacting proteins
