# Supplementary material for: TRIM28-mediated nucleocapsid protein SUMOylation enhances SARS-CoV-2 virulence
Source: Nat Commun. 2024 Jan 4;15:244. doi: 10.1038/s41467-023-44502-6 (PMC10764958; doi:10.1038/s41467-023-44502-6)
Supplement: Supplementary file 5 — Reporting Summary [file 41467_2023_44502_MOESM5_ESM.pdf]

nature portfolio

Corresponding author(s): Fangfang zhou

Last updated by author(s): Nov 23, 2023

Reporting Summary

Nature Portfolio wishes to improve the reproducibility of the work that we publish. This form provides structure for consistency and transparency in reporting. For further information on Nature Portfolio policies, see our Editorial Policies and the Editorial Policy Checklist.

Statistics

For all statistical analyses, confirm that the following items are present in the figure legend, table legend, main text, or Methods section.

n/a	Confirmed	
☐	☒	The exact sample size (*n*) for each experimental group/condition, given as a discrete number and unit of measurement
☐	☒	A statement on whether measurements were taken from distinct samples or whether the same sample was measured repeatedly
☐	☒	The statistical test(s) used AND whether they are one- or two-sided<br>*Only common tests should be described solely by name; describe more complex techniques in the Methods section.*
☒	☐	A description of all covariates tested
☐	☒	A description of any assumptions or corrections, such as tests of normality and adjustment for multiple comparisons
☐	☒	A full description of the statistical parameters including central tendency (e.g. means) or other basic estimates (e.g. regression coefficient) AND variation (e.g. standard deviation) or associated estimates of uncertainty (e.g. confidence intervals)
☐	☒	For null hypothesis testing, the test statistic (e.g. *F*, *t*, *r*) with confidence intervals, effect sizes, degrees of freedom and *P* value noted<br>*Give P values as exact values whenever suitable.*
☒	☐	For Bayesian analysis, information on the choice of priors and Markov chain Monte Carlo settings
☒	☐	For hierarchical and complex designs, identification of the appropriate level for tests and full reporting of outcomes
☒	☐	Estimates of effect sizes (e.g. Cohen's *d*, Pearson's *r*), indicating how they were calculated

Our web collection on statistics for biologists contains articles on many of the points above.

Software and code

Policy information about availability of computer code

Data collection	1. Enzyme linked Immunosorbent Assay data were collected using BioTek Synergy Neo2 Multi-Mode Reader.<br>2. Immunofluorescence images were acquired using a ZEISS LSM 880 confocal microscope.<br>3. qPCR were performed using Bio-Rad CFX-96 Touch.<br>4. Mass Spectrometry was performed on a Q Exactive HF-X Hybrid Quadrupole-Orbitrap Mass Spectrometer .<br>5. The interaction between protein-protein or protein-RNA was detected by the MONOLITH NT.115 system (NanoTemper Technologies).<br>The software used for data collection was described in "Methods". No special code were used for data collection.
Data analysis	GO enrichment analysis were conducted by the R package (clusterProfiler, v3.17) .GO enrichment results were further analyzed with DAVID for generating interactive graphs.<br>Images were recorded by Zeiss LSM880 confocal microscope system(ZEN 2.3(blue edition)).<br>Mass Spectrometry data was analyzed by Maxquant (v1.6.17.0) and Perseus (v1.6.1.3).<br>The occupied area, equivalent diameter of droplets, the percentage of cells harboring puncta, the ratio of puncta-like fluorescence intensity, and the PLA-detected proximity (PROX) complexes intensity were quantified by Image J (1.53h) .<br>The FRAP data were fitted to a single exponential model using the GraphPad Prism 9.<br>Statistical analysis was performed with Graph Pad Prism 9.

For manuscripts utilizing custom algorithms or software that are central to the research but not yet described in published literature, software must be made available to editors and reviewers. We strongly encourage code deposition in a community repository (e.g. GitHub). See the Nature Portfolio guidelines for submitting code & software for further information.

Data

Policy information about availability of data

All manuscripts must include a data availability statement. This statement should provide the following information, where applicable:

- Accession codes, unique identifiers, or web links for publicly available datasets
- A description of any restrictions on data availability
- For clinical datasets or third party data, please ensure that the statement adheres to our policy

A full data availability statement is included in the manuscript. The information of antibodies, plasmids, qPCR primer sequences, labeled RNA sequences, and other regents used in this study were provided in Methods. The data that support the findings of this study are available from the corresponding author upon request. The MS/MS raw data generated in the current study are available in the ProteomeXchange Consortium via the iProX partner repository with the dataset identifier PXD046366 (https://www.iprox.cn//page/project.html?id=IPX0007355000). All other data are provided in the Article, Supplementary and Source data files. Source data are provided with this paper.

Research involving human participants, their data, or biological material

Policy information about studies with human participants or human data. See also policy information about sex, gender (identity/presentation), and sexual orientation and race, ethnicity and racism.

Reporting on sex and gender	N/A
Reporting on race, ethnicity, or other socially relevant groupings	N/A
Population characteristics	N/A
Recruitment	N/A
Ethics oversight	N/A

Note that full information on the approval of the study protocol must also be provided in the manuscript.

Field-specific reporting

Please select the one below that is the best fit for your research. If you are not sure, read the appropriate sections before making your selection.

☒ Life sciences ☐ Behavioural & social sciences ☐ Ecological, evolutionary & environmental sciences

For a reference copy of the document with all sections, see nature.com/documents/nr-reporting-summary-flat.pdf

Life sciences study design

All studies must disclose on these points even when the disclosure is negative.

Sample size	For FRAP experiments, luciferase reporter assay, real-time quantitative PCR, plaque assay, microscale thermophoresis assay, tandem mass spectrometry, n = 3 independent samples were used for sample size. For in vitro phase separation (droplet formation) assay, n = 6 independent images were used for quantifying the occupied area. For puncta observation in cellulo, n = 6 independent images were used for quantifying the percentage of cells harboring puncta , or 10 independent cells for quantifying the ratio of puncta-like fluorescence intensity to background. The number of the independent experiments was indicated in each figure legend. This selection was made to produce reproducible results with a significance level of less than 0.05 and a power exceeding 90%.<br>Based on our extensive experience with animal model and endpoints, we usually use at least 6 mice per group as described in the manuscript, to make sure the generation of reproducible results with a significance level of less than 0.05 and a power exceeding 90%.
Data exclusions	No data were excluded from analysis.
Replication	All the findings were reliably reproduced in multiple independent experiments, which were indicated in text and figure legend. For cell and biochemical experiments, our data represent at least three independent experiments with similar results. For mice experiments, data are representative of at least two independent experiments with similar results. We also used different assays and readouts to confirm our findings in different way.
Randomization	All the cell experiments in vitro were performed by plating the cells in independent dishes or wells, and randomly assigned to control or experimental groups without bias introduced. Mice were randomly allocated to each group prior to any treatment.
Blinding	Investigators were blinded to group allocation during the data collection and analysis wherever possible. Some In vitro biochemical experiments like WB, were not conducted in a blinded manner, because the experiments were planned and performed by the same investigator. The quantification for FRAP experiments, luciferase reporter assay, real-time quantitative PCR, plaque assay, microscale thermophoresis assay, in vitro phase separation, puncta observation in cellulo was performed blindly. For mice experiment, the investigators were blinded to group allocation prior to the data collection and/or analysis.

Reporting for specific materials, systems and methods

We require information from authors about some types of materials, experimental systems and methods used in many studies. Here, indicate whether each material, system or method listed is relevant to your study. If you are not sure if a list item applies to your research, read the appropriate section before selecting a response.

Materials & experimental systems

n/a	Involved in the study
☐	☒ Antibodies
☐	☒ Eukaryotic cell lines
☒	☐ Palaeontology and archaeology
☐	☒ Animals and other organisms
☒	☐ Clinical data
☒	☐ Dual use research of concern
☒	☐ Plants

Methods

n/a	Involved in the study
☒	☐ ChIP-seq
☒	☐ Flow cytometry
☒	☐ MRI-based neuroimaging

Antibodies

Antibodies used

The antibodies used in this study include: rabbit monoclonal anti-SUMO-2/3 (Cell Signaling, 4971; 1:1,000 for IB), mouse monoclonal anti-SARS2-NP (ABclonal, A20142; 1:2,000 for IB, 1:200 for PLA), rabbit monoclonal anti-SARS2-NP (Abcam, ab271180; 1:200 for IP), rabbit monoclonal anti-ACE2 (Abcam, ab108252; 1:1,000 for IB), rabbit monoclonal anti-TRIM28 (ABclonal, A19568; 1:2,000 for IB, 1:100 for IP/PLA), mouse monoclonal anti-Flag (M2) (Sigma, F3165; 1:2,000 for IB, 1:200 for PLA), rabbit polyclonal anti-HA (Y11) (Santa Cruz, sc-805; 1:2,000 for IB, 1:200 for PLA), rabbit monoclonal anti-HA (12CA5) (Santa Cruz, sc-57592; 1:2,000 for IB), rabbit polyclonal anti-Myc (A-14) (Santa Cruz, sc-789; 1:2,000 for IB, 1:200 for PLA), mouse monoclonal anti-Myc (9E10) (Santa Cruz, sc-40; 1:2,000 for IB, 1:200 for IP), mouse monoclonal anti-His (H-3) (Santa Cruz, sc-8036; 1:2,000 for IB), mouse monoclonal anti-β-actin (Sigma, A1978; 1:2,000 for IB), rabbit monoclonal anti-β-tubulin (Cell Signaling, 2146; 1:2,000 for IB), rabbit polyclonal anti-VSV-G (ABGENT, AP1016a; 1:1,000 for IB), rabbit monoclonal anti-spike protein (ABclonal, A20022; 1:200 for IHC), and HRP-conjugated secondary antibodies (Cell Signaling, 7076 (anti-mouse IgG) or 7074 (anti-rabbit IgG); 1:10,000 for IB).

Validation

Antibodies were chosen based on the available literature. Quality of the antibodies used in the study was tested by manufacturer or relevant references cited on the manufacturer's website. Additional information on validation can be found on the manufacturers' websites.
rabbit monoclonal anti-SUMO-2/3 (Cell Signaling, 4971; 1:1,000 for IB): https://www.cellsignal.com/products/primary-antibodies/sumo-2-3-18h8-rabbit-mab/4971
mouse monoclonal anti-SARS2-NP (ABclonal, A20142; 1:2,000 for IB, 1:200 for IP/PLA): https://abclonal.com.cn/catalog/A20142
rabbit monoclonal anti-TRIM28 (ABclonal, A19568; 1:2,000 for IB, 1:100 for IP/PLA): https://abclonal.com.cn/catalog/A19568
mouse monoclonal anti-Flag (M2) (Sigma, F3165; 1:2,000 for IB, 1:200 for PLA): https://www.sigmaaldrich.com/SG/en/product/sigma/f3165
rabbit polyclonal anti-HA (Y11) (Santa Cruz, sc-805; 1:2,000 for IB, 1:200 for PLA): https://www.scbt.com/p/ha-probe-antibody-y-11/
rabbit monoclonal anti-HA (12CA5) (Santa Cruz, sc-57592; 1:2,000 for IB): https://www.sebt.com/p/ha-probeantibody-12ca5#:—:text=See%20product%20citations%20%28198%29%20HA-Tag%20Antibody%20%2812CA5%29%20is,containing%20the%20HA%20tag%20by%20WB%20and%20IP.
rabbit polyclonal anti-Myc (A-14) (Santa Cruz, sc-789; 1:2,000 for IB, 1:200 for PLA): https://www.scbt.com/p/c-myc-antibody-a-14/
mouse monoclonal anti-Myc (9E10) (Santa Cruz, sc-40; 1:2,000 for IB): https://www.scbt.com/p/c-myc-antibody-9e10
mouse monoclonal anti-β-actin (Sigma, A1978; 1:2,000 for IB): https://www.sigmaaldrich.com/SG/en/product/sigma/a1978
rabbit monoclonal anti-β-tubulin (Cell Signaling, 2146; 1:2,000 for IB): https://www.cellsignal.com/products/primary-antibodies/btubulin-antibody/2146
rabbit polyclonal anti-VSV-G (ABGENT, AP1016a; 1:1,000 for IB): https://www.abeepta.com/products/AP1016a-VSV-g-Tag-Antibody
rabbit monoclonal anti-spike protein (ABclonal, A20022; 1:200 for IHC): https://abclonal.com.cn/catalog/A20022
HRP-conjugated secondary antibodies (Cell Signaling, 7076 (anti-mouse IgG): https://www.cellsignal.com/products/secondaryantibodies/
anti-mouse-igg-hrp-linked-antibody/7076. or 7074 (anti-rabbit IgG); 1:10,000 for IB): https://www.cellsignal.com/products/secondary-antibodies/anti-rabbit-igg-hrp-linked-antibody/7074

Eukaryotic cell lines

Policy information about cell lines and Sex and Gender in Research

Cell line source(s)

The HEK293T/F, HeLa, A549, Vero E6 and CaCo-2 cell lines were obtained from American Type Culture Collection. RAW264.7 cells were kindly provided by Stem Cell Bank, Chinese Academy of Sciences. Human bronchial epithelial cell line 16HBE was obtained from Sigma (SCC150). Mouse embryonic fibroblast (MEF) cells were isolated from embryo of C57BL/6 mice at E14.5 organogenesis. Peritoneal macrophages were harvested from the C57BL/6 mice 4 days after injection of thioglycolate.

Authentication

The HEK293T/F, HeLa, A549, Vero E6 and CaCo-2 cell lines have been purchased from ATCC. RAW264.7 cells were kindly provided by Stem Cell Bank, Chinese Academy of Sciences. 16HBE was obtained from Sigma. The identity

	of cell lines have been authenticated by related organizations using short tandem repeat analysis. All cell lines were frequently checked for cellular morphologies, growth rates, and functions.
Mycoplasma contamination	We confirm that all cell lines were negative for mycoplasma contamination at regular intervals.
Commonly misidentified lines (See ICLAC register)	No commonly misidentified cell lines were used in this study.

Animals and other research organisms

Policy information about studies involving animals; ARRIVE guidelines recommended for reporting animal research, and Sex and Gender in Research

Laboratory animals	Six- to eight-week old male C57BL/6 mice were purchased from Beijing Vital River Laboratory Animal Technology Co., Ltd. TSix- to eight-week old male hACE2 transgenic mice (C57BL/6-Tgtn (CAG-human ACE2-IRES-Luciferase-WPRE-polyA) Smoc, NM-TG-200002) were purchased from Shanghai Model Organisms Center, Inc. (http://www.modelorg.com/portal/article/index/id/9665/post_type/3.html). Mice were maintained under specific-pathogen-free conditions in the animal. The animal room has a controlled temperature (18-23°C), humidity (40-60%), and a 12 light/12 dark cycle.
Wild animals	No wild animals were used in this study.
Reporting on sex	Only male mice were used in this study, and sex was not considered in study design.
Field-collected samples	No field-collected samples were used in this study.
Ethics oversight	All animal experiments were approved and reviewed by the Institutional Committee for Animal Welfare of Soochow University (for biosafety-level-2 animal experiments), or by the Ethics Committee of ZSSOM of Sun Yat-sen University (for biosafety-level-3 animal experiments).

Note that full information on the approval of the study protocol must also be provided in the manuscript.

Plants

Seed stocks	N/A
Novel plant genotypes	N/A
Authentication	N/A
